# Video Illustrations of Techniques and Strategies for Adult and Pediatric Spinal Deformity Surgery

**DOI:** 10.3171/2020.1.FocusVid.Intro

**Published:** 2020-01-01

**Authors:** Justin S. Smith, Christopher I. Shaffrey, Michael Wang, Mohamad Bydon, Lawrence Lenke

**Affiliations:** 1 Department of Neurosurgery, University of Virginia, Charlottesville, Virginia;; 2 Departments of Neurosurgery and Orthopedic Surgery, Duke University, Durham, North Carolina;; 3 Department of Neurosurgery, University of Miami, Miami, Florida;; 4 Department of Neurosurgery, Mayo Clinic, Rochester, Minnesota; and; 5 Department of Orthopedic Surgery, Columbia University, New York, New York

Spinal deformity includes a broad range of pathologies and has an impact across the continuum of life, from early-onset scoliosis (EOS) in a young child to degenerative scoliosis with sagittal malalignment in the elderly. How these patients are managed clinically and surgically has advanced remarkably over recent decades, but the field remains in evolution. Surgical treatments are often complex, include techniques that are challenging, and continue to have relatively high complication rates. This issue of *Neurosurgery Focus: Video* provides 11 videos that illustrate a variety of spinal deformity treatment techniques and revision strategies.

Three-column osteotomies are among the most complex procedures for spinal deformity correction. Two videos focus on these osteotomies, including the use of vertebral column resection (VCR) for severe kyphosis correction by Gupta and the use of a special hinged table for closure of pedicle subtraction osteotomies (PSOs) by Chang and colleagues. Two additional videos describe approaches to help avoid the need for these major osteotomies, including the application of hyperlordotic anterior lumbar interbody fusion (ALIF) cages by Marino and colleagues and the performance of minimally invasive lateral anterior column release (ACR) by Ohiorhenuan and colleagues.

Two videos describe the relatively new technique of oblique interbody fusion, one demonstrating its application to iatrogenic deformity (Wilkerson et al.) and the other incorporating the use of navigation (Chang et al.). Two additional videos focus on revision techniques, one addressing the challenging problem of proximal junctional kyphosis (PJK) (McClendon et al.) and the other demonstrating the revision of surgically treated adolescent idiopathic scoliosis (AIS) in a patient who developed symptomatic degenerative changes later in life (Burke et al.). High cervical pathologies are discussed in two videos that address posterior treatment for os odontoideum (Zhao et al.) and techniques for vertebral artery mobilization at the atlantoaxial level (McDowell et al). Navarro-Ramirez and colleagues show a novel growing rod technique to treat EOS.

Collectively, the videos in this issue illustrate techniques and strategies to address a broad range of deformities, from EOS in infants and children to fixed sagittal plane deformities in adults. Modern surgical treatments for spinal deformity continue to evolve, and it is in large part by sharing these skills and advancements through the literature and video media that this knowledge is disseminated. We offer our sincere appreciation to those who submitted works for this special issue and hope that the audience finds these videos useful as they endeavor to care for their spinal deformity patients.

